# Gene Expression Profiles Resulting from Stable Loss of p53 Mirrors Its Role in Tissue Differentiation

**DOI:** 10.1371/journal.pone.0082494

**Published:** 2013-11-28

**Authors:** Oliver Couture, Eric Lombardi, Kendra Davis, Emily Hays, Nalini Chandar

**Affiliations:** Department of Biochemistry, Chicago College of Osteopathic Medicine, Midwestern University, Downers Grove, Illinois, United States of America; Hertie Institute for Clinical Brain Research, University of Tuebingen., Germany

## Abstract

The tumor suppressor gene p53 is involved in a variety of cellular activities such as cellular stress responses, cell cycle regulation and differentiation. In our previous studies we have shown p53’s transcription activating role to be important in osteoblast differentiation. There is still a debate in the literature as to whether p53 inhibits or promotes differentiation. We have found p53 heterozygous mice to show a p53 dependency on some bone marker gene expression that is absent in knockout mice. Mice heterozygous for p53 also show a higher incidence of osteosarcomas than p53 knockout mice. This suggests that p53 is able to modify the environment within osteoblasts. In this study we compare changes in gene expression resulting after either a transient or stable reduction in p53. Accordingly we reduced p53 levels transiently and stably in C2C12 cells, which are capable of both myoblast and osteoblast differentiation, and compared the changes in gene expression of candidate genes regulated by the p53 pathway. Using a PCR array to assay for p53 target genes, we have found different expression profiles when comparing stable versus transient knockdown of p53. As expected, several genes with profound changes after transient p53 loss were related to apoptosis and cell cycle regulation. In contrast, stable p53 loss produced a greater change in MyoD and other transcription factors with tissue specific roles, suggesting that long term loss of p53 affects tissue homeostasis to a greater degree than changes resulting from acute loss of p53. These differences in gene expression were validated by measuring promoter activity of different pathway specific genes involved in differentiation. These studies suggest that an important role for p53 is context dependent, with a stable reduction in p53 expression affecting normal tissue physiology more than acute loss of p53.

## Introduction

The tumor suppressor gene, *p53*, has been widely investigated as a transcription factor involved in multiple cellular processes including DNA damage, hypoxia, and cell cycle regulation [1-5]. In addition to its more traditional roles in stress responses and cell cycle regulation, p53 also plays a role during development and cellular differentiation [6,7]. In our laboratory, we have found the osteocalcin gene, which codes for a protein associated with terminal osteoblast differentiation, to be regulated by p53 in committed osteoblast cells [8]. Other studies have found several more tissue specific genes to be directly regulated by wild type p53, such as MyoD, a late signal of skeletal muscle differentiation [9,10]. While there are several studies indicating a role for p53 in cell differentiation, not all of them are consistent for a role promoting differentiation. In neuronal cells, localization of p53 to mitochondria is important in determining if cells undergo differentiation or apoptosis [3]. In mesenchymal stem cells, p53 appears to keep progenitor cells from entering their cell type committed pathways by negatively regulating key differentiation transcription factors, such as Pparγ for adipogenic differentiation, Myocd for myofibroblast differentiation, and Runx2 for osteogenic differentiation [11]. In embryonic stem cells, however, the role of p53 is more complex. p53 represses both Nanog and 4-Oct, both of which repress differentiation in embryonic stem cells. p53, however, can also activate the Wnt family of genes, resulting in inhibition of differentiation of stem cells [12]. So while p53 appears to inhibit early differentiation and keep cells in a more stem like state, late differentiation of certain cell fates are in part reliant upon the activity of p53.

The importance of tissue specificity with regard to p53 function is further supported by studies carried out in p53 transgenic mice [13,14]. In addition to an increased incidence in tumor formation in p53 null mice, there is also evidence of a spectrum of developmental defects such as exencephaly, defective spermatogenesis and skeletal abnormalities in a small percentage of the animals [15]. A high proportion of the tumors in the p53 null mice are lymphomas followed by osteosarcoma and soft tissue tumors [13]. In p53 heterozygous mice, the tumor spectrum is altered with a higher incidence of osteosarcomas and soft tissue tumors than lymphomas . This could mean that there are functionally redundant pathways that make up for loss of p53 during development. However, its presence albeit in small amounts, is able to influence osteoblast function underscoring the important of p53 in maintaining normal bone remodeling and preventing neoplastic transformation. Clinically osteosarcoma show heterogeneous tumor types thought to arise from the disruption of osteoblast differentiation at different times [16]. Since p53 appears to normally inhibit entry into osteoblast differentiation, but is needed for terminal differentiation, it is not surprising that a partial disruption of p53 promotes osteosarcoma development.

Since p53 responds to multiple stimuli and can affect multiple, different cellular processes, changes in p53 expression will affect cells differently depending on whether the change is transient or stable. In this study we test this by using shRNA interference to reduce p53 levels in a bipotential cell line, C2C12, transiently and compared the changes in gene expression of genes involved in p53 pathways with a stable reduction of p53. We found greater changes in expression of MyoD and of other transcription factors with tissue specific roles in the stable p53 knockdown cells, which suggests that long term partial loss of p53 expression affects tissue homeostasis to a greater degree than changes resulting from transient loss of p53. Additionally, we show differences in the promoter activities of genes involved in differentiation between stable and transient loss of p53. Together this points toward the role of p53 as being context dependent, with a stable loss affecting cellular differentiation more than a transient loss.

## Materials and Methods

### Cell line and growth conditions

 In order to study the role of p53 in the differentiation of mesenchymal stem cells, the bi-potential cell line C2C12 (ATCC, cat no. CRL-1772) was selected because it can differentiate into myotubes and can be induced to osteoblasts, depending on the culture media [17,18]. These cells were maintained in DMEM/F-12 media with 10% fetal bovine serum and incubated in T-75 flasks at 37°C in 5% CO_2_/95% air. 

### Knockdown of p53 in C2C12 cells by shRNA

The p53 HuSH-29 shRNA plasmids (Origene®, cat. no. TF500002), were used to create transient and stable knockdown lines of p53 within the C2C12 cells. We used three different shRNA sequences to knockdown p53. Control cells received in a similar sized non effective scrambled sequence (TR20003). SuperFect (QIAGEN, cat. no. 301305) was used for both transient and stable transfections according to manufacturer’s protocol. Additionally, for obtaining stable clones, cells were selected post transfection using puromycin. Single cell clones were expanded, passaged and characterized before use. 

### RNA Extraction, Purification, and Quantification

To determine the changes at the transcriptional level, RNA was isolated either using TRI Reagent (Sigma, cat. no. T9424) or the QIAGEN RNeasy Mini Kit (cat. no. 74104) prep system depending on the assay used. Genomic contamination of the RNA was removed using the Ambion DNA Free kit (Ambion, cat. no. AM1906). DNA free RNA was quantified using the Nanodrop2000 (Thermo Scientific, cat. no. ND-2000).

### RT-PCR

Semiquantitative RT-PCR was used to measure the relative expression of p53 after shRNA introduction as well as, the various bone and muscle markers.. RNA was reverse-transcribed using the QIAGEN OneStep RT-PCR kit (cat. no. 210210) using equivalent amounts of RNA. The following primers were ordered from QIAGEN, and used according to their protocol: Gapdh (cat. no. QT01658692) and p53 (cat. no. QT00101906). To measure the intensity of the resulting PCR, the samples were run on a 1% agarose gel containing ethidium bromide and the image captured on a Kodak system. The image was then quantified using the UnscanIt™ software and the relative quantification was calculated using Gapdh to normalize the expression.

### PCR Array

In addition to looking at the various differentiation markers, a more in depth analysis of pathway perturbations was also assayed using the QIAGEN/SABiosciences p53 Signaling Pathway PCR Array (SABiosciences, cat. no. PAMM-027A). Genes included on the array are direct targets of p53, regulators of p53, or genes in a p53 pathway that are downstream of p53, and cover many of the different pathways p53 is found to affect, such as apoptosis, differentiation, and cell cycle regulation, as well as control genes for normalization, genomic DNA contaminating and RT efficiency. The RNA obtained for a stable line of shRNA plasmid 34 (S34), 36 (S36), as well as, a transient transfection created from plasmid 36 (T36) and a control line of C2C12 cells was made using QIAGEN RNeasy Mini Kit (cat. no. 74104) prep was first quantified using the Nanodrop 2000, then 1.5 µg of total RNA was reverse-transcribed using the RT^2^ First Strand Synthesis Kit (SABiosciences, cat. no. 330401) according to the standard protocol. A master mix using the RT^2^ SYBR Green/ROX kit (SABioscineces, cat. no. 330520) was made for each sample and then the array was then loaded and ran on an ABI Prism 7300 Real Time PCR System, using the settings as defined by SABiosciences for their arrays. Once run, the data was normalized using the included genes on the array, then fold changes were calculated by using the standard 2^-ΔΔCt^ method, using the array of the control cells as the second Δ.

### Quantitative RT-PCR

In order to validate results of the e PCR arrays, several genes that underwent changes in expression were selected, and quantitative real-time PCR (QPCR) was performed (see Results section) on triplicate samples of S36, T36, and control C2C12 cells. Specific primers for Realtime analyses are as follows. p53 (QIAGEN, cat. no. QT00101906), MyoD (Integrated DNA Technologies (IDT), Coralville, IA, forward 5’-GTTCTTCACGCCCAAAAGATG-3’, reverse 5’-GGACAGTTGGGAAGAGTGTCATT-3’), Pten (QIAGEN, cat. no.QT00141568), p300 (QIAGEN, cat. no.QT00291900), Rb1 (IDT, forward 5’-TCGATACCAGTACCAAGGTTGA-3’, reverse 5’-ACACGTCCGTTCTAATTTGCTG-3’), Bag1 (IDT, forward 5’-GAGATGGTCCAGACGGAGGA-3’, reverse 5’-ACCTTGCTGTGGGGTAACAA-3’), Casp2 (IDT, forward 5’-TGGTGTAGATGGCAAACTGCT-3’, reverse 5’-CCACGACATGCTTGGATGAAG-3’), Casp9 (IDT, forward 5’-TGGCTCCTGGTACATCGAGA-3’, reverse 5’-TTCGCAGAAACAGCATTGGC-3’), Mcl1, (IDT, forward 5’-TAAGGACGAAACGGGACTGG-3’, reverse 5’-TTCTGATGCCGCCTTCTAGG-3’), Numb (IDT, forward 5’-GTACCTCGGCCACGTAGAAG-3’, reverse 5’-TCCCGTTTTTCCAAAGAAGCC-3’), and Prkca (IDT, forward 5’-GGCAACATGGAACTCAGGCA-3’, reverse 5’-TCTGTCCAGGTTGTTGGATGG-3’), with 18S (QIAGEN, cat. no. QT02448075) used as a normalizer. These primers were used in an assay using SYBR Green QPCR on duplicate test samples. Samples of RNA free of DNA contamination were reverse transcribed using the Ambion High Capacity cDNA kit (Ambion, cat. no. 4368814). A master mix containing the Applied Biosystems *Power*SYBR® Green 2x Master Mix (Applied Biosystems, cat. no. 4367659), an equal amount of the cDNA reaction, and water to make 25 µl volume samples were created for each gene. The mix was then aliquoted into a 96 well plate, primer added, and ran on an Agilent 7300 Real Time PCR System. Fold changes were calculated using the 2^-ΔΔCt^ method using 18S as the first Δ with the control samples as the second Δ.

### Protein Extraction and Quantification

C2C12 clones with the different p53 shRNAs were grown to confluency for stable lines or two days post transfection for transient transfected lines. Total protein was isolated from each plate using M-PER mammalian protein extraction reagent (Pierce, cat. no. 78503) with supplemented Roche Complete ULTRA Tablets protease inhibitor (Roche, cat. no. 50892791001). Protein concentration was measured using Bradford assay (Thermo Scientific, cat. no. 1856209). Following isolation and quantification, the proteins were subjected to western blot analyses. A 25 or 50 μg fraction of each protein extract was separated on a SDS PAGE gel, then electro transferred onto presoaked PVDF paper (Bio-Rad, cat. no. 162-0177). The membranes were then blocked with 5% milk protein in PBS/T, and the primary antibody for either p53 (Cell Signaling, cat. no. 2524S) or β-actin (Sigma, cat. no. A2066) was added and allowed to incubate overnight at 4°C. The membrane was then washed with PBS/T three times, and HRP conjugated secondary antibodies to mouse or rabbit IgGs (Thermo Scientific, ImmunoPure Antibody) was added, washed again, and exposed using the ECL Western blotting reagents (AmershamPharmicia Biotech, cat. no. RPN2209). The image was then captured on a Kodak system and UnscanIt™ was used to obtain quantification.

### Reporter Assays

Several gene specific reporter assays were carried out to examine the effect of transient and stable p53 knockdown. Our reporter constructs consisted of mainly luciferase reporters but some of them were Chloramphenicol Acetyl Transferase (CAT) based reporters. Specific details for both assays are provided below. 

When stable p53 knockdown lines were used, cells received just the reporter construct. When the assay was carried out in cells with transient knockdown of p53, we either used a specific shRNA construct or a mixture of three p53 shRNA constructs. Specific details are provided in figure legends. Control cells received a scrambled sequence of a similar length in the same plasmid backbone (for knockdown of p53) and/or the empty vector (reporter assays). 

### Luciferase Assays

Forty-eight hours after transfection, they were trypsinized, pelleted, and then resuspended in serum free media. An aliquot was used to count cells for normalization, the rest was assayed using Promega’s Bright GloTM Luciferase Assay Kit (Promega, cat. no. E2610). Equal volumes of the resuspended cells and Bright-GloTM were mixed and incubated for 2 minutes. A Turner Designs TD20/20 Luminometer was used to record the luciferase activity for each sample. Samples were read for 10 seconds, each after a two second delay. Luciferase readings for each of the samples were then normalized according to the number of cells/ml. All measurements were carried out on triplicate samples and experiments were repeated at least thrice.

### Measurement of p53 functional activity

In order to evaluate the functional activity of p53 we transfected cells with, pG13-luc [19], a plasmid containing 13 canonical p53 binding site driving a luciferase reporter Control cells received a scrambled sequence (MG-15-luc) [19].

### Chloramphenicol Acetyl Transferase Assays

Forty-eight hours after transfection the cells were lysed and equal amounts of protein were used to measure chloramphenicol acetyltransferase (CAT) activity using n-Butyryl CoA and ^14^C Chloramphenicol. The product was extracted with xylene and radioactivity measured using a liquid scintillation counter. All measurements were carried out on triplicate samples and experiments were repeated at least thrice.

### Developmental Pathways tested

Because p53 is involved in the development and differentiation of C2C12 cells into later, more committed cell types, the promoters of certain genes representing various pathways were assayed for activity in the stable and transient p53 knockdowns to test to see if transient or stable loss of p53 had different effects upon the expression of bone or muscle markers. The notch pathway was assayed using the Hey1-luc plasmid [20]. In addition, the Vitamin D pathway was assayed using the VTTK-CAT plasmid [21]. Also, the activity on the promoters of a bone and muscle specific gene, osteocalcin and muscle creatine kinase, respectively, were analyzed using the CAT based pOSCAT3 [22] and p3300MCKCAT [23] plasmids, respectively. All assays were carried out in triplicate and repeated at least thrice. 

### Statistics and bioinformatics

All statistics were done in GraphPad Prism 5 (Microsoft), using either unpaired t-tests or one-way ANOVA with Tuckey correction for multiple testing and post hoc tests, and requiring a p < 0.05 for significance. To visualized the difference between how T36 and S36 differ when compared to control cells, clustering was done using Cluster3 [24] by using the fold change values calculated as above for the corresponding arrays, then adjusting the data by using the negative inverse for any value lower than one. These values were then mean centered before clustering. Hierarchical clustering was run using Euclidean distance as the similarity metric, and using centroid linkage as the method. The resulting clustering was viewed in TreeView [25], which was also used to create a heat map.

## Results

### Generation of cells with stable and transient reduction of wild type p53

**Figure 1:**
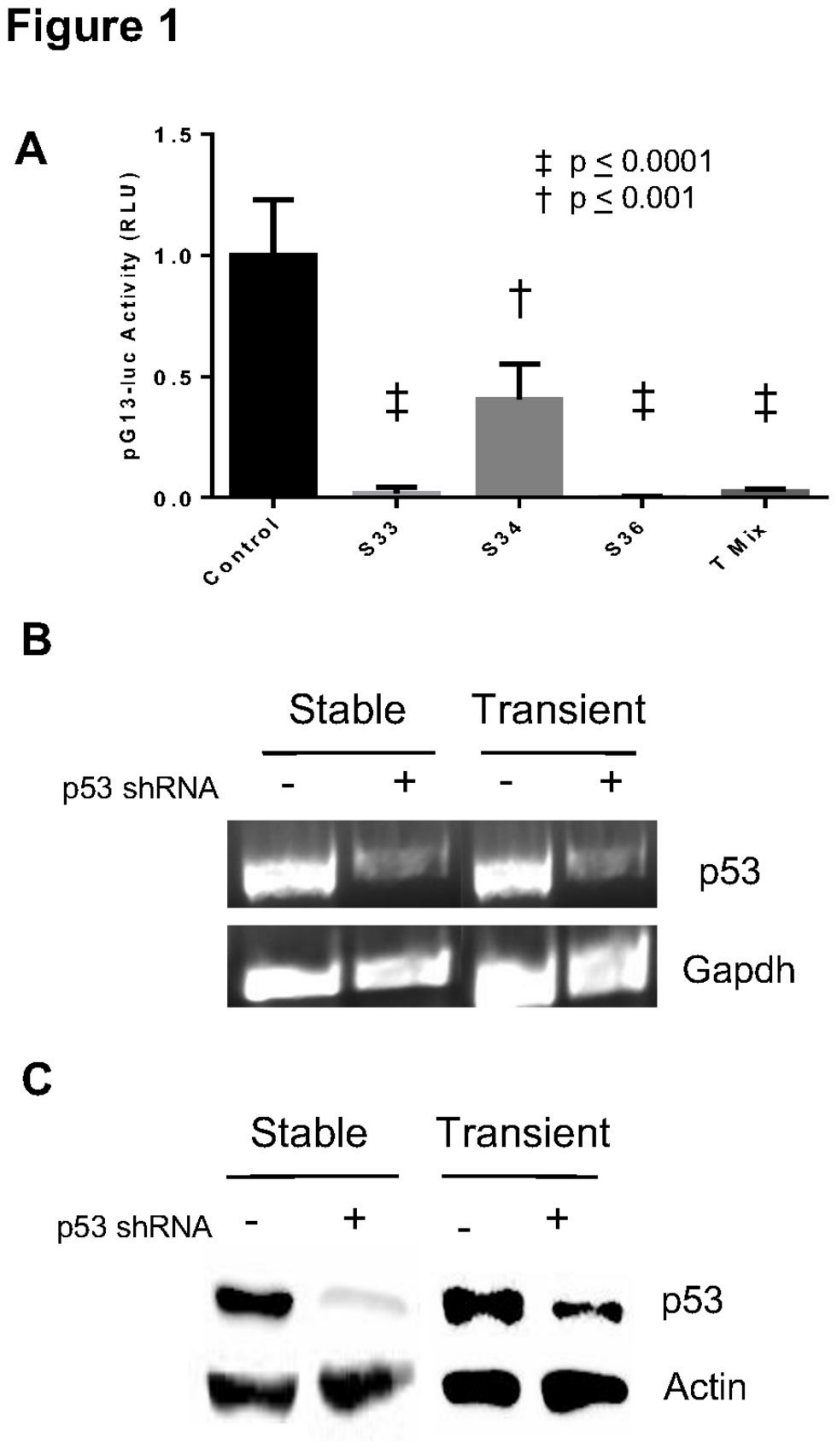
Confirmation of stable and transient knockdown of p53 (A) Transcriptional activity of p53 is reduced with reduction in p53 levels Mean + S.E.M. of relative luciferase units (RLU) reported for stable (S) knockdown of p53 using shRNA plasmids and a transient knockdown using a mix of all three plasmids (T Mix) when compared to controls receiving scrambled shRNA sequences. (**B**) RT-PCR of p53 from a stable knockdown (S33), a transient knockdown (T33) and control (scrambled shRNA). (**C**) Immunoblot of p53 expression in a stable (S36) line and after transient knockdown of p53 (T36). The same blot was stripped and reprobed with anti-actin antibody. Similar results were obtained in three independent experiments and representative data are shown.

In order to create a stable knockdown of p53, we isolated puromycin resistant single cell clones after transfecting various p53 shRNA constructs in a plasmid conferring puromycin resistance into C2C12 cells. Individual clones were expanded and characterized. The same constructs were also used for transient reduction of p53, but no selection was performed and cells were harvested after 48 hours for RNA and protein. Our intention was to generate cells with a similar knockdown in p53 using the same shRNA construct. In order to rule out possible dosage dependent effects between and among the different constructs and type of tranfections, several methods were used to assess the knockdown Therefore in addition to determining extent of p53 knockdown at the transcriptional and translational level, we tested the level of p53 functional activity using a construct containing 13 copies of a p53 binding sequence [19]. This construct was transiently transfected into stable p53 knockdown lines we had generated and p53 functional activity was measured 48 hours later. In the case involving transient transfections both constructs (reporter and shRNA) were transfected together. As seen in Figure 1A, different levels of knockdown of p53 activity was achieved with the different p53 shRNA constructs when compared to cells receiving a non- specific scrambled sequence. Among the stable lines two out of three lines showed greater knockdown of p53 activity. A mixture of all three shRNAs also produced an effective knockdown. As clone 33 and clone 36 demonstrated effective knockdown of p53 function we further proceeded to use the same shRNA constructs to transiently reduce p53 in C2C12 cells. A representative RT-PCR of a stable clone 33 line is shown compared to stable control created with a scrambled sequence. Similarly using the same constructs in transient transfections we show a similar knockdown of p53 (Figure 1A lanes 3 and 4). In Figure 1C we show representative results from use of a different shRNA that was used to create clone 36. In all these cases we obtained knockdown of 65-90% of endogenous p53 levels after analyses of band intensities. More importantly we chose to further analyze samples where the extent of knockdown in stable and transient cultures was comparable using the same shRNA sequence. 

### Realtime PCR arrays of p53 target genes show differences between stable and transient loss of p53

**Figure 2:**
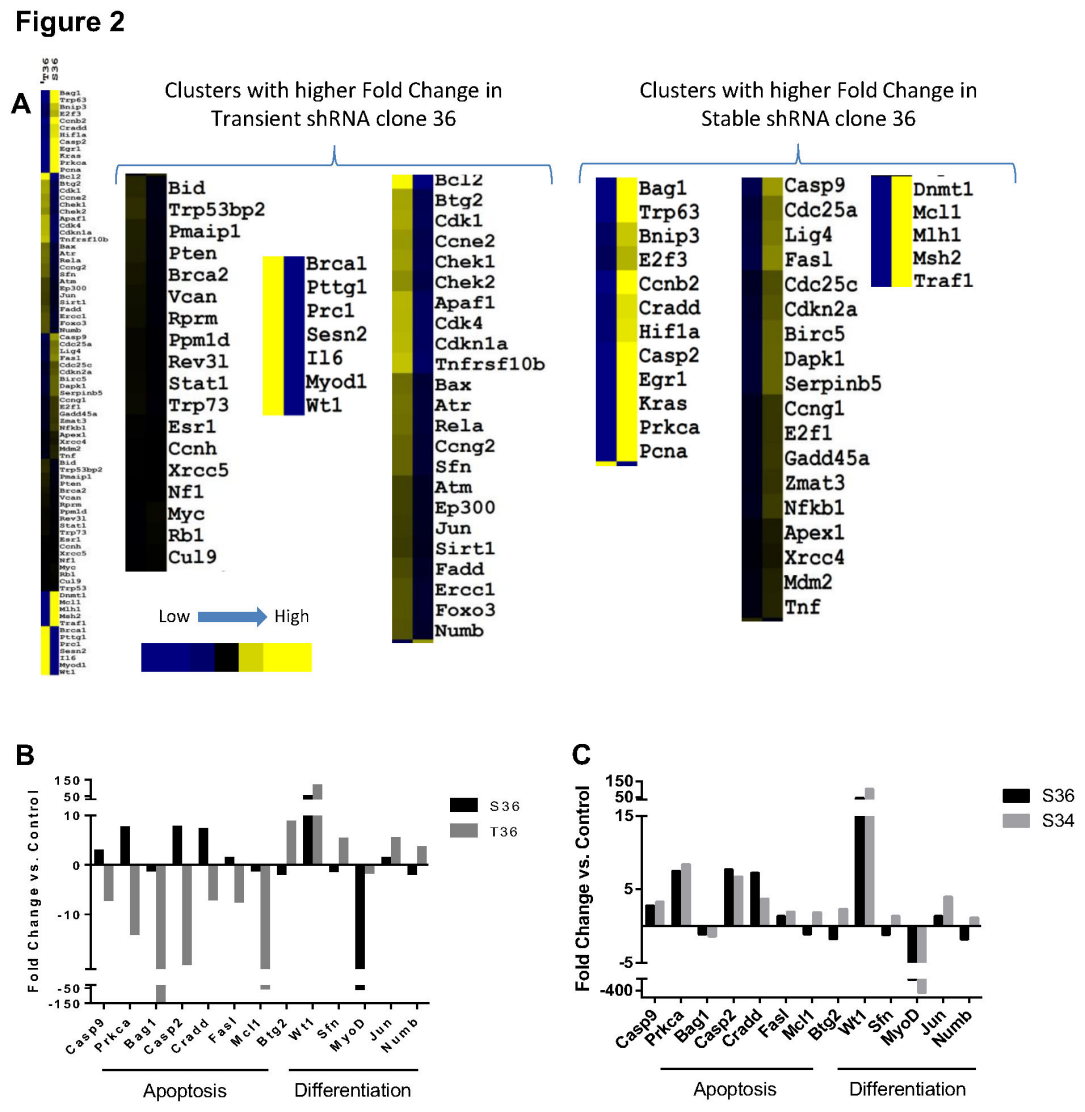
Gene expression o f p53 target genes after p53 loss. (A) Heat map generated by centroid linkage of the Euclidian distance of mean centered fold change values of transient transfection of shRNA plasmid 36 (T36) and stable line created with plasmid 36 (S36) using a control line (created with scrambled shRNA) as a common divisor, 2^-((T36-Normalizers) – Control)^ compared to 2^-((S36-Normalizers) – Control)^, (n = 1). The negative inverse used for fold changes between 0 and 1. Clusters were then divided into two categories, those with higher expression in T36 and those with higher expression in S36. (**B**) Fold change values plotted for genes involved in apoptosis or differentiation with a fold change between treated and control cells greater than two (**C**) Comparison of stable clones of varying p53 expression. Plot of the same gens as in (B) but between S36 and a stable line created with clone 34 (S34) show stable p53 knockdown irrespective of the level of knockdown, have a much more similar expression pattern than seen between transient and stable.

In order to look for more global differences between acute loss, from the transient knockdown, and a long term loss of p53 the SABioscience Q-PCR array was used to assay the changes in genes related to p53. We chose to compare RNA from stable and transient knockdown created with the shRNA (S36 vs. T36). RNA from both these cells was first compared to control cells receiving a scrambled shRNA sequence. As expected, many of the targets showed changes with loss of p53 expression. Hierarchical clustering of the expression changes between the two lines are shown as compared to control cells. Two types of change were observed one where genes are up-regulated in the acute loss compared to the long term loss of p53, and a second cluster with genes up-regulated in long term p53 loss when compared to the acute loss. (Figure 2A). As expected, when compared to control, the genes that are anti-apoptotic are down-regulated with transient loss of p53 (Casp2, Casp9, Cradd, Fasl, Bag1, etc.) (Figure 2B). In contrast, these genes are slightly up-regulated when p53 is knocked down stably. Similarly, the genes classified as differentiation related (Btg2, Jun, MyoD, Numb, Sfn, and Wt1), with the exception of MyoD, are all up-regulated when p53 was knocked down transiently. In stable p53 knockdown lines they show variable expression (Figure 2B). This may relate to the fact that some of them are tissue specific with little relevance to osteoblasts or myocytes. 

### Dosage of p53 does not alter target gene expression in stable p53 knockdown cells

Some of our previous studies demonstrated that the amount of p53 within osteoblasts might affect cell proliferation and differentiation variably [26-28]. Since one of our stable clones (clone 33) showed a smaller knockdown of p53 activity (~50%) we compared this line to our stable line 36 which has a greater knockdown of p53 activity (~ 80%) to see if the difference in p53 level affected its target genes is a dissimilar manner in C2C12 cells. A duplicate gene array to the one described in Figure 2A was done using a control and the two stable p53 knockdown lines. Our results showed very little if any variation between the two stable lines with differing dosage (Figure 2C). This suggests that irrespective of the dosage, stable loss of p53 affects target genes differently from that produced by transient p53 loss. 

### Validation of array results with realtime PCR

**Figure 3:**
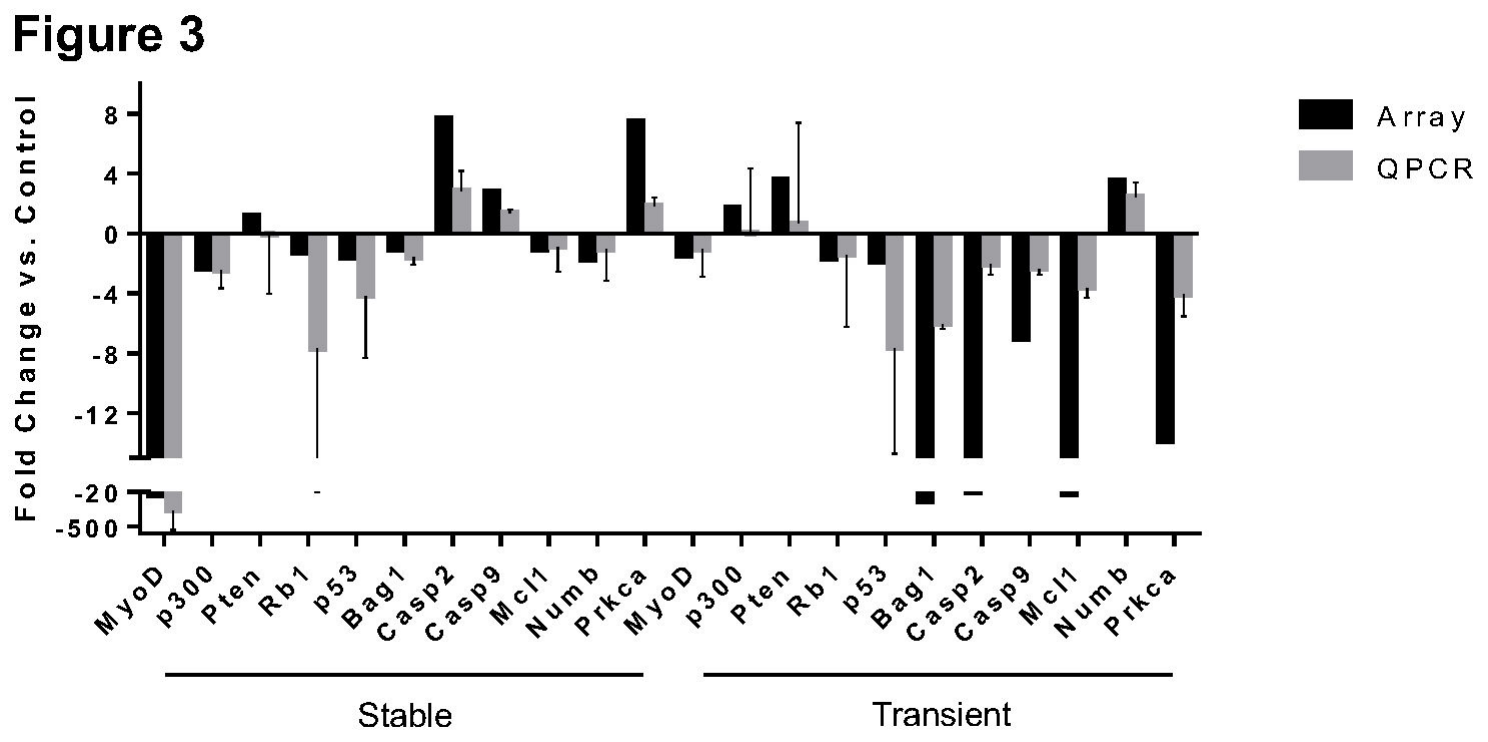
Array Confirmation. Confirmation of targets from gene array by Quantitative PCR verifies array expression values. QPCR of selected genes that showed variation in expression in arrays were selected and tested with three independently isolated single cell clones using the same shRNA as the array(S36), Transient transfections were carried out in triplicate with the same shRNA , and control cells received the scrambled control plasmid (mean + S.E.M., n = 3) for verification. For each gene, the change in expression matched that of the array in both types of transfections.

Several genes belonging to apoptosis and differentiation related pathways showing alterations in the array were chosen for validation using realtime PCR. For this analysis we created new stable lines with p53 knockdown using the same shRNA. Transient knockdown of p53 was also carried out in triplicate. RNA from these newly generated cell lines were compared to results obtained from the gene array. As shown in Figure 3, the quantitative PCR results agreed with the array results in the direction of the change in expression. As expected of independent samples there was a variation in the extent of change between samples, but most importantly the directionality of the change was maintained for all of the genes tested, thus validating the results of the array. The variation seen in the case of some of the genes may relate to differences in the level of p53 knockdown as three independent clones/transfections were compared to the array. Additionally they may represent changes in steady state levels of expression dependent on the status of the cells at the time of RNA isolation. 

### Developmental pathway markers differ in their responses to acute and stable p53 loss

**Figure 4:**
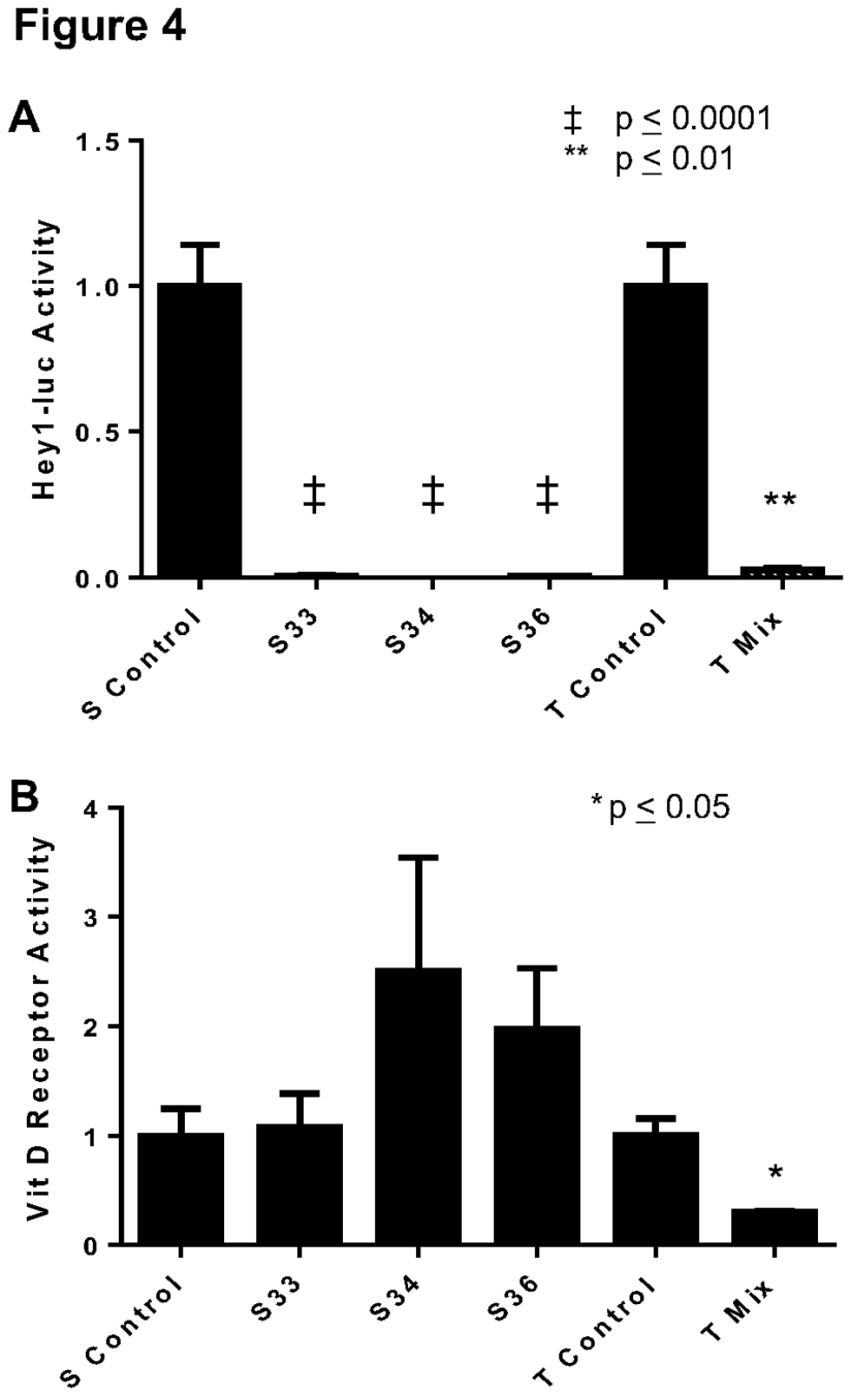
Activities of Notch and Vitamin D receptor pathways after p53 loss. (**A**) Hey1-luc reporter shows a significant reduction in Notch signaling activity of S33, S34, and S36 when compared to controls. Similar results are seen for T Mix when compared to control. (**B**) Vitamin D receptor reporter plasmid shows non-significant changes with long term p53 reduction. However, a loss was observed in T Mix cells when compared to control. Three novel clones (n = 3) were measured in triplicate, and the mean of each triplicate was used to calculate the presented mean ± S.E.M. for each group.

In addition to the expression data, we wanted to determine if specific pathways related to differentiation are activated differently dependent on whether p53 loss was stable or transient. The notch pathway is known to be important for tissue differentiation and has been shown to be important for both myoblast and osteoblast differentiation [29,30]. When the differences in the promoter activity of a direct target in notch signaling, Hey1 [31],between stable and transient p53 knockdowns were compared to control cells, there was a dramatic reduction, regardless of the type of transfection (p < 0.001; Figure 4A). We also tested the change of activity of vitamin D receptor which is important for both myoblast and osteoblast differentiation [32,33]. In contrast to the Hey1 reporter, the vitamin D pathway showed a different response depending on the length of p53 knockdown. A stable reduction in p53 resulted in a non-significant increase seen (p > 0.05; Figure 4B), whereas, an acute loss of p53 resulted in a significant decrease in activity of the vitamin D receptor promoter (p < 0.05; Figure 4B).

**Figure 5:**
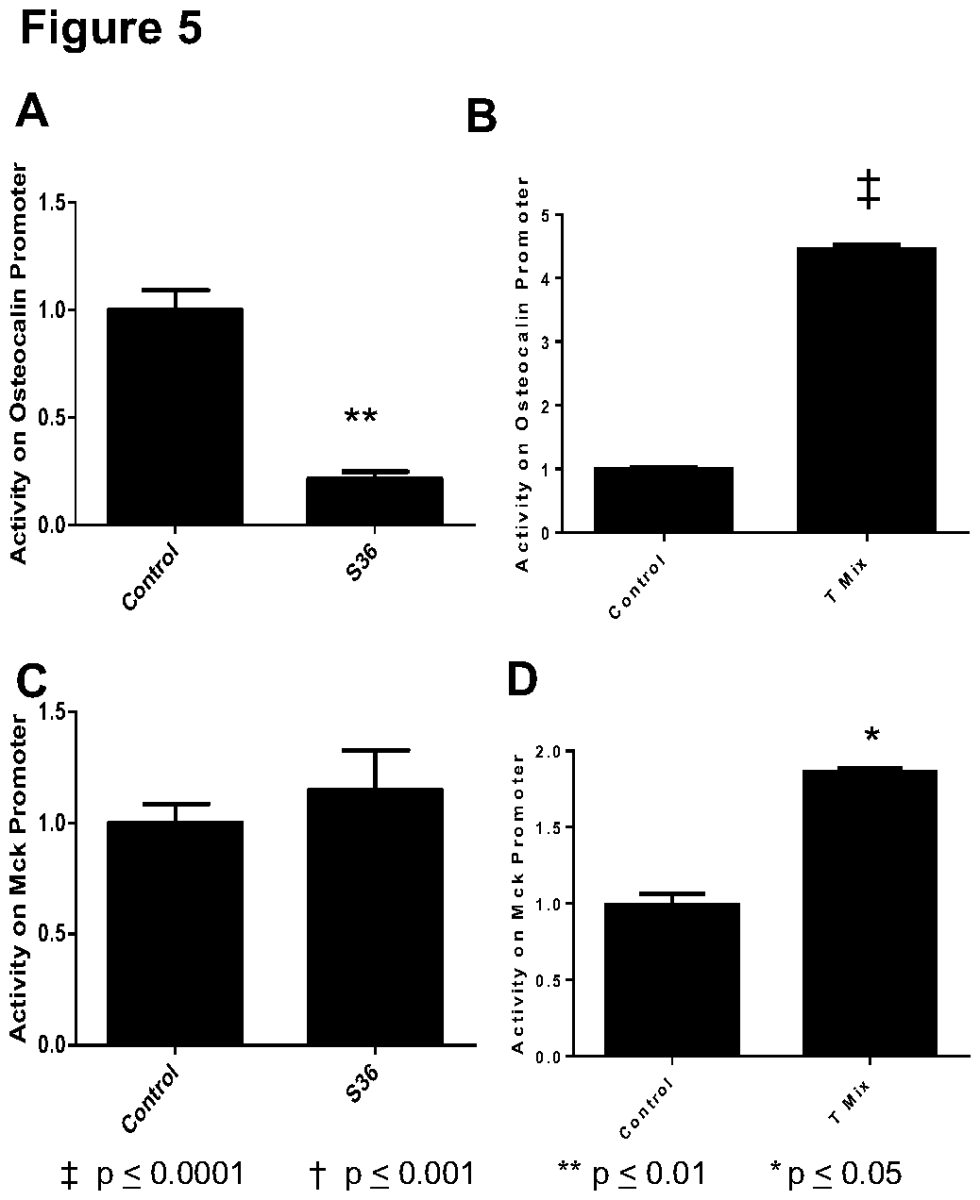
Bone and muscle differentiation markers show different patterns based on the type of transfection. (**A**) Osteocalcin gene promoter shows loss of activity between S36 and control cells created with a scrambled shRNA sequence. (**B**) Conversely, in T Mix samples, an increase in the activity on the osteocalcin promoter when compared with control cells receiving scrambled shRNA. (**C**) Muscle creatine kinase (MCK) gene promoter activity, showed no difference in activity when S36 is compared to control cells. (**D**) Promoter activity increased in T Mix when compared to control cells. Three novel clones (n = 3) were measured in triplicate each, using the mean to calculate the presented mean ± S.E.M. for each group.

Since the C2C12 cells can differentiate into either myocytes or osteoblasts, we tested the promoter activity of the genes Muscle Creatine Kinase (MCK), a late marker of myogenic differentiation, and osteocalcin, a late marker of osteoblast differentiation. When p53 is stably reduced the loss results in a significant decrease in activity of the osteocalcin promoter (p < 0.001; Figure 5A). With transient loss, osteocalcin promoter activity is increased (p < 0.001; Figure 5B). In contrast to osteocalcin, the MCK promoter did not change after stable loss of p53 when compared to control. (p > 0.05; Figure 5C). However, like osteocalcin, the promoter activity of MCK was also elevated initially by the knockdown of p53 (p < 0.01; Figure 5D). This is in agreement with our previous work demonstrating a requirement of p53 function for expression of the osteocalcin gene [8]. 

## Discussion

p53 is a transcription factor that regulates a number of different genes affecting cellular conditions ranging from proliferation, apoptosis, and DNA repair to cellular metabolism and senescence. While a role for p53 in cellular differentiation is well accepted, there is still a debate on whether p53 facilitates or opposes differentiation. This stems from the fact that loss of p53 during development is associated with little or no effect on tissue differentiation [13]. In the case of bone, complete loss of p53 results in normal bone development and increased osteoblast differentiation [26]. We have previously shown that in the presence of reduced dosage of wild type p53, osteoblast differentiation is p53-dependent [26]. This suggests that redundant pathways may exist, but in the presence of at least partial amounts of p53, the dosage of p53 is a critical determinant of osteoblast function. However, targeted knockout of p53 in bone has consistently shown this gene to have an inhibitory effect on osteoblast proliferation and differentiation [34]. In our studies we have used committed osteoblasts to demonstrate that osteocalcin, a bone matrix protein expressed in advanced osteoblast differentiation, is only produced when p53 is present [27,28]. We have also shown the osteocalcin gene is a direct transcriptional target of p53 [8]. Osteoblast differentiation progresses through stages of proliferation and expression of several matrix proteins followed by quiescence and expression of osteocalcin and generation of a mineralized matrix [35]. Osteoblasts arise from common mesenchymal progenitors and progress through commitment to the osteoblast lineage to preosteoblasts and osteoblasts. It is therefore possible that p53 has distinct stage specific roles during development and differentiation. In the context of osteosarcomas, p53 is generally absent due to a p53 gene rearrangement [36] and replacement of wild type p53 in these cells is associated with inhibition of tumor growth and the appearance of a calcified matrix [28]. Osteosarcoma tumors also generally do not express osteocalcin [37]. Since bone remodels through life, it is possible that the presence of p53 is important to maintain homeostasis in post natal bone. This suggests that p53 may influence the environment within bone to allow orderly differentiation in addition to maintaining a balance of cell proliferation and cell death. 

In an attempt to separate the different roles of p53 we studied the short and long term effect of p53 loss on its target genes. In this study we utilized C2C12, a bipotential cell line capable of differentiation towards myoblast and osteoblast, to determine the effect of transient and stable knockdown of p53 using RNA interference. An acute loss of p53 lowered the mRNA expression for a number of genes related to apoptosis. This is expected, as one of the major roles of p53 is in regulating apoptosis, a function that represents an immediate response to prevent damaged DNA and or cells from survival. However, these same genes show a small increase in expression after long term loss of p53; perhaps as an adaptation to reduced p53 levels. Specifically, members of the caspase family of genes, Casp2 and Casp9, as well as the Casp2 assembly protein Cradd, are all down-regulated with an acute loss of p53, whereas they appear to be up-regulated when p53 is reduced for the long term. It is also possible that the change seen in caspases with stable p53 loss may reflect other functions unrelated to apoptosis as has been seen for some cell types [38-40]. Similarly, the expression of Prkca was also greatly reduced in the transient lines and elevated in the stable lines. While Prkca function in apoptosis is well understood [41] and is represented as an inducer of apoptosis in this gene array (http://www.sabiosciences.com/rt_pcr_product/HTML/PAMM-027A.html), several roles have been attributed to Prkca and its class of proteins in signaling and cell proliferation [41]. Therefore, the response seen after stable p53 loss may be symbolic of change in growth control. Irrespective of the reasons, the differences in responses appear to be consistent with the type of p53 loss. 

The apoptotic pathway is not the only pathway that appears to differ according to the length of p53 knockdown. Genes involved in p53 related differentiation pathways varied in expression based on the type of p53 loss. MyoD and Numb are well known differentiation and cell fate related genes [42,43]. Validation of these genes with realtime PCR show that these changes are consistent with the type of p53 loss. Our array data show Btg2, Wt1, MyoD, Jun, and Numb all have a higher expression in an acute loss of p53. This may reflect a generalized stress response produced as a result of a sudden decrease in p53 expression. Numb is a repressor of the Notch signaling pathway [44], and its elevation after transient loss of p53 may relate to p53’s role as an enhancer of notch signaling [45]. However, under conditions of stable loss such an effect is absent and may reflect other complementary roles of Numb in regulating p53 function [46] MyoD has the largest decrease after stable loss of p53, consistent with evidence that it is a p53 regulated gene [47] and myogenesis represents the default pathway in C2C12 cells [17]. While the changes in the other genes are difficult to extrapolate to C2C12 differentiation, it is clear that these differentiation associated genes are affected markedly only after chronic loss of p53 suggesting that the deficiency of p53 has brought about a change in the environment within these cells. 

We studied lineage specific, as well as, lineage independent signaling pathways that might be affected by the knockdown of p53 in C2C12 cells. We investigated activity of the Notch pathway and did not find differences between stable and transient knockdown as in both cases this pathway was depressed with loss of p53. In a genome wide study carried out to determine p53 regulators, the notch pathway, especially Hey1, was found to be members of an evolutionarily conserved network governing p53 function [48]. It was interesting to note that our studies show a mutual regulation with loss of p53 affecting the Notch pathway. In the case of vitamin D receptor activity, while a significant effect was not seen with chronic loss of p53, a transient loss produced a reduction in the receptor’s function. Vitamin D and its receptor are known to have potent genomic as well as non-genomic action in myocytes and on calcium and phosphate metabolism [49]. Our results probably represent these complex pathways that are affected with p53 loss. We also studied activities of two genes of advanced differentiation in C2C12 cells, one for each of the two common lineages. MCK is seen late during myocyte differentiation and osteocalcin is a late marker of bone differentiation. One of the immediate changes after loss of p53, as measured with the transient samples, was an up-regulation in activity of these genes. However, after a long term loss of p53 only osteocalcin activity was decreased. This is consistent with other studies that have shown p53 to be necessary for osteogenic reprogramming of skeletal muscle committed cells [11]. 

These studies indicate that while the role of p53 is important to protect cells from damage by regulating key apoptotic genes, p53 has an additional role in maintaining tissue homeostasis. This may be especially important in osteoblasts since bone remodels throughout life. The presence of p53 may be critical to allow for orderly progression of the different steps of the differentiation process. p53 is a direct regulator of matrix proteins osteopontin and osteocalcin [8,50]. Osteocalcin expression coincides with a decrease in proliferation and matrix deposition and the initiation of the mineralization process. Loss of p53 in tumors may therefore produce a constitutive proliferative state with the lack of osteocalcin, a feature commonly seen in osteosarcomas. This study shows a distinct difference in expression of differentiation markers with long term loss of p53 may result from the loss of ability of p53 to maintain the balance between regulatory factors, and or acutely sense and modify the environment for orderly differentiation.
